# Complications and compliance in professionally-managed and self-managed contact lenses compared with non-contact lens wearers

**DOI:** 10.1371/journal.pone.0308538

**Published:** 2024-09-06

**Authors:** Liat Gantz, Barry A. Weissman, Reut Ifrah

**Affiliations:** 1 Department of Optometry and Vision Science, Hadassah Academic College, Jerusalem, ISRAEL; 2 Southern California College of Optometry at Marshall B. Ketchum University, Fullerton, CA, United States of America; 3 Stein Eye Institute and Department of Ophthalmology, David Geffen School of Medicine at UCLA, Los Angeles, CA, United States of America; University of Houston College of Optometry, UNITED STATES OF AMERICA

## Abstract

**Purpose:**

To test the impact of professional management of soft contact lens wear on symptoms and ocular complications.

**Methods:**

Subjective symptoms and ocular complications of soft CL users who did not seek professional follow-up care (self-managed, SM), were compared to users who were prescribed CLs and their care professionally managed in optometry practices (PM), and to a control group of non-CL wearers. Habitual visual acuity, subjective dry-eye symptoms, and corneal abnormalities were assessed in all participants. CL wearers filled-out a usage habits questionnaire, and their CL fit was assessed. Outcomes were compared using Kruskal-Wallis and Chi Squared tests.

**Results:**

The SM, PM, and non-CL wearers cohorts included 127 (mean age:24.3±5.1, median:23, range:16–45 years,104 female), 132 (mean age:25.5±6.2, median:23, range:18–43 years,103 female), and 56 (mean age:22.3±3.5, median:21, range:18–39 years,36 female) participants, respectively. Meibomian gland dysfunction grade (p = 0.004, p<0.0001), limbal redness (both p = 0.04), corneal neovascularization (both p = 0.003), and papillary conjunctivitis (p<0.0001,p = 0.005) were significantly worse in SM CL wearers compared with both the non-CL wearers and PM CL wearers, respectively. Conjunctival staining was significantly worse in the SM cohort compared with the PM cohort (p = 0.01). 38.6% of the SM compared with 22.8% of the PM CL wearers, had an inappropriate refractive correction (p = 0.006). SM CL wearers wore CLs significantly more years (mean and median 1 year,p = 0.008), for more daily hours (mean and median of 2 hours,p<0.00001), and tended to nap or sleep with their CLs compared with the PM CL wearers (47 vs. 29,p = 0.02). The cohorts did not differ in their subjective symptoms.

**Conclusions:**

Complications are significantly more prevalent in SM CL wearers compared with PM CL wearers, and SM CL wearers tend to wear CLs with incorrect powers, and are less compliant with napping or sleeping with the CLs compared with PM CL wearers. These findings emphasize the importance of fitting, patient education and follow-ups in CL wearers.

## Introduction

There are approximately 140 million contact lens (CL) wearers worldwide; CLs are used for refractive error correction, myopia control, therapeutic purposes, and cosmetic indications [1].

Although the majority of CL users wear their lenses successfully and comfortably, numerous studies and case reports document patients who suffer ocular complications during wear [2–11]. These complications may result in reduced wearing time, discontinuation of CL wear, need for emergency eye care, and on rare occasions, vision loss [12].

Research has rightfully been focused on the sight-threatening complications of microbial keratitis [7–10] and corneal neovascularization [10, 11]. Most CL complications, however, are not immediately sight-threatening and often may be remedied with appropriate clinical management if diagnosed early in their courses [3]. If left untreated, however, even minor complications may eventually lead to discomfort, tissue morbidity, discontinuation of wear, or perhaps even loss of vision and permanent disability [13]. Corneal neovascularization is one example of an initially asymptomatic complication which can eventually lead to vision loss if left undiagnosed and untreated, [11] but other complications such as CL papillary conjunctivitis, corneal infiltrates, etc. can also become problematic if undiagnosed and untreated [13].

Patient education, both during the initial fitting of the CLs and at subsequent follow-up visits, promotes compliant behavior which includes hand washing, correct lens handling and cleaning [14] and case care including timely case replacement [15]. Non-compliant behavior is associated with an increased risk of complications, ranging from decreased comfort and vision to more serious or sight-threatening infectious events [14].

Microbial keratitis, [2] papillary conjunctivitis, [4] neovascularization, [4, 10, 11] corneal aberrations, [4] corneal infiltrates, [4] corneal edema, [5] superficial punctate staining, [5] central circular clouding, [6] corneal ulcers, [7] ulcerative keratitis, [8, 9] corneal opacification, [11] and many other complications [3] have been linked with CL wear [2–11]. Thus, CL wear can be considered a risk factor for complications that can be detected and prevented early with professional evaluations of asymptomatic CL wearers. Hence CL wearers may similarly benefit from routine asymptomatic professional eye evaluations rather than only seek care when symptomatic [16, 17].

In the United States, a CL prescription is legally required to purchase CLs. CL prescriptions are usually valid for one year following the examination date. Therefore, all patients in the United States, including asymptomatic ones, are required to undergo a professional eye exam to acquire new CL prescriptions on a yearly basis, allowing for the diagnosis and management of complications detected in asymptomatic CL wearers. In contrast, in other parts of the world, such as Israel, CLs may be legally purchased over-the-counter. In these countries, CL wearers are self-managed: they decide for themselves whether or not they should seek periodic professional care. Asymptomatic patients in these countries may therefore, for financial or convenience reasons, acquire new CLs without seeking professional care, in some cases for many years.

We hypothesized that self-managed CL users would have lower compliance to guidelines and more complications than professionally- managed CL users. We evaluated the usage practices, visual outcomes, subjective symptoms, and ocular complications in soft CL wearers who were not professionally followed (self-managed, SM) to users who were prescribed CLs and their care professionally managed in optometry practices (PM). To verify that these complications are due to contact lens wear and not due to other factors, these two cohorts were further compared with a control cohort of non-CL wearers.

## Methods

The study adhered to the tenets of the Declaration of Helsinki and was approved by the internal ethics committee at Hadassah Academic College (HAC), Jerusalem Israel. Participants signed a written statement of informed consent after receiving an oral explanation prior to participation.

CL wearers were recruited between June 06, 2017 and July 09, 2020. Control non-CL wearers were recruited between September 24, 2019 and September 03, 2021. The control non-CL wearers were primarily drawn from a separate study (Ifrah et al. [18]). However, the present study includes two additional control participants who were not analyzed in the previous study due to missing outcome measures.

### Participants

In this study, 315 participants between the ages of 16–45 were recruited from the clinics at the HAC Department of Optometry, from two private practices, and from advertisements posted at HAC and on social media.

Participants were divided into three cohorts; self-managed (SM) CL wearers, professionally-managed CL wearers (PM), and self-reported “healthy” controls who never used CLs. The inclusion of a control group ensures that the ocular complications observed only amongst CL wearing cohorts can be attributed to CL use rather than other factors [19].

Inclusion criteria for CL wearers included at least one year of soft CL use, at least five days of CL wear per week and at least five hours of daily CL wear (based on Machalinska et al. [20]). The PM CL cohort included participants who had been seen for CL adjustment or aftercare with a slit lamp examination by an ophthalmologist or optometrist within the preceding year based on self-report. This inclusion criteria was based on the recommendation that the time interval between routine professional aftercare visits for soft CLs is 12 months [21]. The SM CL cohort included participants had not been seen by an ophthalmologist or optometrist for a CL adjustment or follow-up for corneal health assessment with a slit lamp biomicroscope, over the course of at least the last year.

Exclusion criteria for all cohorts included known ocular infection, inflammation, allergy, past ocular surgery, ocular diseases such as keratoconus, systematic diseases (e.g., hypertension, diabetes mellitus, ischemic heart disease), or use of medications that might affect the tear film (such as antihistamines and hormones). Previous or current RGP lens wearers, pregnant or lactating women were also excluded.

### Procedures

All examinations took place in the CL clinic at the HAC Department of Optometry, in a designated examination room. Control non-CL wearers who required a visual correction, wore their habitual correction for the monocular LogMAR visual acuity measurement. Visual acuities of emmetropic control participants were measured without any visual aid.

Participants arrived wearing their habitual CLs for at least one hour prior to the study visit. After measuring habitual monocular LogMAR visual acuity, CL wearers filled out a questionnaire pertaining to their CL modality, replacement schedule, solutions, habits of wear and hygiene (S1 Appendix) [13]. Usage habits were evaluated by assessing of years of CL wear, daily hours of CL wear, napping or sleeping with the CLs, case care (changing the solution in the case every day, replacement of the case every three months and solution issue), and washing hands before handling CLs. Participants that reported napping or sleeping with their CLs, or that they did not wash hands prior to CL handling, or did not change the solution daily, or the CL case every three months were considered non-compliant.

Both eyes of each participant were evaluated and only the right eye was included in the analysis, due to the likely correlation between the eyes [22]. Slit-lamp biomicroscopy (Huvitz Slit Lamp HS-5000) was performed to assess abnormalities including meibomian gland dysfunction, blepharitis, conjunctival redness, limbal redness, corneal neovascularization, epithelial microcysts, corneal edema, corneal staining, conjunctival staining, papillary conjunctivitis, corneal infiltrates, corneal ulcer, endothelial polymegethism/blebs, corneal distortion, superior limbic keratoconjunctivitis. The abnormalities graded binary 0/1 (0 if not present, 1 if present). Meibomian gland dysfunction was graded 0–4 based on Efron’s grading scale [23]. The lens fit was assessed by examining the base curve (good/ steep/flat), lens overall diameter (good/small/large), and optical power (good/weak/strong).

Participants were asked to report if they suffer from symptoms of dry eye on a binary scale (1-yes, 0- no).

### Statistical analysis

Demographic data was evaluated using descriptive statistics. The means, standard deviations, and medians of the study outcomes from the participants’ right eyes were calculated.

The normality of the outcome measures was assessed using the Kolmogorov-Smirnov test. Three cohorts were compared (PM CL wearers, SM CL wearers, and non- CL wearing controls).

For outcome variables with binary scales, a Pearson Chi-squared test was applied to compare the cohorts. Outcome variables with a continuous scale (0–4) were compared using the Kruskal-Wallis test with post-hoc analysis.

Using G-Power software (ver. 3.1.9.7; Heinrich-Heine-Universität Düsseldorf, Düsseldorf, Germany, https://www.psychologie.hhu.de/arbeitsgruppen/allgemeine-psychologie-und-arbeitspsychologie/gpower), [24] the *minimum* sample size required when comparing three cohorts that are not normally distributed, for a power (1-ß) of 80%, error probability of 5% (α error 0.05) and an effect size (d) of 0.55, is 53 participants.

Statistical analysis was performed with Microsoft Office Excel (Microsoft.com) and IBM SPSS Statistics (version.27, ibm.com). P-values < 0.05 were considered significant.

## Results

### Participants

Of the 315 participants (243 (77.1%) female, age range: 16–45 years, mean age: 24.4±5.5 years), 127 were PM CL wearers (81.9% female, age range: 16–45, mean age: 24.3±5.1 years), 132 SM CL wearers (78% female, age range: 18–43, mean age: 25.5±6.2 years) and 56 control non- CL wearers (64.3% female, age range: 18–39, mean age: 22.3±3.5 years). The demographic data for the cohorts is detailed in Table 1. A chi-square test of independence showed a significant difference between the three cohorts (PM, SM and controls) in the number of females (*X*^2(1)^ = 6.38, p = 0.01, *X*^2(2)^ = 6.93, p = 0.03, respectively) with post hoc (Bonferroni) analysis revealing significantly more females in the PM and SM cohorts compared to the control cohort. Further, there were significant differences in the ages the three cohorts (PM, SM and controls, p = 0.003) with the PM and SM cohorts older by an average of two years compared with the control cohort.

**Table 1:** Demographic data, distance visual acuity and refractive outcome measures of control, non-contact lens wearers (NCL), professionally- managed (PM) contact lens wearers, and self-managed (SM) contact lens wearers included in the study.

Data		NCL wearers	PM	SM	P
**Participants** **(N = 315)**		56	127	132	0.03 Post hoc: M> NCL, p = 0.01 SM> NCL, p = 0.04
**Female**		36 (64.3%)	104 (81.9%)	103 (78.0%)	
**Age (years)**	**Mean ± SD**	22.3±3.5	24.3±5.1	25.5±6.2	0.003 Post hoc: PM>NCL, p = 0.006 SM > NCL, p = 0.0006
	**Median**	21.0	23.0	23.0	
	**Quartiles (Q1, Q3)**	20,23	21,26	21,30	
	**Range**	18–39	16–45	18–43	
**Distance VA (LogMAR)**	**Mean ± SD**	0.03 ± 0.1	0.05 ± 0.1	0.07 ± 0.1	0.03 Post-hoc: NCL > SM, p = 0.002
	**Median**	0.00	0.00	0.10	
	**Quartiles** **(Q1, Q3)**	(0.0,0.1)	(0.0,0.1)	(0.0,0.1)	
	**Range**	0.0–0.2	0.0–0.3	0.0–0.3	
**Sph (D)**	**Mean ± SD**	-0.77±1.7	-3.11±2.8	-3.44±2.2	<0.00001 Post-hoc: PM>NCL, p<0.001 SM>NCL, p<0.001
	**Median**	0.00	-3.00	-3.25	
	**Quartiles** **(Q1, Q3)**	(-0.9,0.0)	(-4.25,-1.75)	(-4.5,-2.0)	
	**Range**	-7.00-+1.50	-10.75- +4.75	-11.50- +3.00	
**Cyl (D)**	**Mean ± SD**	-0.19±0.4	-0.51±0.7	-0.17±0.4	0.07
	**Median**	0.00	0.00	0.00	
	**Quartiles** **(Q1, Q3)**	(-0.3,0.0)	(-0.81,0.0)	(0.0,0.0)	
	**Range**	-2.50–0.00	-2.50–0.00	-1.75–0.00	

The number of participants, number of female participants, age, distance visual acuity (VA), and spherical and cylindrical refractive components of the spectacle refraction (excluding the contact lens prescription) of the experimental cohorts of the control, non-contact-lens wearers (NCL), professionally managed (PM) and self-managed (SM) contact-lens wearers included in the study. The p-values represent results of the chi-square test of independence applied to compare between the number of males and females in the cohorts and the Kruskal-Wallis test comparing the ages, visual acuity and refractive components of the cohorts. Post hoc Bonferroni comparisons of significant tests appear below.

As seen in Table 1, the three cohorts differed significantly in their distance VA (p = 0.03) and their refractive spherical component (P<0.00001), but not in their refractive cylindrical component (p = 0.07).

Post-hoc comparisons (Bonferroni) found that the non-CL wearers had significantly better distance VA compared with the SM CL wearers by a mean of 0.04 LogMAR and a median of 0.1 LogMAR. However, the distance VA was not significantly different between the non-CL wearers and PM CL wearers, or between the SM and PM CL wearers.

Post-hoc comparisons (Bonferroni) also found that the non-CL wearers had significantly lower refractive spherical component values compared with both the PM (mean difference: 2.34 D, median difference: 3.00 D, Table 1) and SM CL wearers (mean difference: 2.67 D, median difference: 3.25 D, Table 1). The PM and SM cohorts did not differ significantly in their refractive spherical component values (p = 0.44).

The CL modality of all CL wearers is detailed in Table 2. There was no significant difference in the CL modality between the PM and SM cohorts (*X*^2(1)^ = 0.81, p = 0.67, Table 2).

**Table 2:** Contact lens modality and fitting characteristics.

	PM (N = 127)	SM (N = 132)	P value
**Contact lens modality**			
**Daily**	50 (39.4%)	45 (34.1%)	0.67
**Bi-weekly**	9 (7.1%)	11 (8.3%)	
**Monthly**	68 (53.5%)	76 (57.6%)	
**Fitting characteristics**			
**Inappropriate optical power**	29 (22.8%)	51 (38.6%)	0.006
**Inappropriate base curve**	25 (19.7%)	37 (28.0%)	0.12
**Inappropriate lens overall diameter**	22 (17.3%)	31 (23.5%)	0.22

The distribution of CL types used by the professionally-managed (PM) and self-managed (SM) cohorts in this study appear in the upper portion of the table. The *X*^2^ statistic showed no significant difference between the CL types used by the PM and SM cohorts. The contact-lens optical power, base curve and overall diameter in the PM and SM; cohorts, with the p-values of the *X*^2^ statistic that was employed to compare between the cohorts (third column) appear in the lower portion of the table.

### Fitting characteristics

The fitting characteristics of the CLs are tabulated in Table 2. The SM CL wearers were significantly more likely than the PM CL wearers to have incorrect optical power (*X*^2(1)^ = 7.57, p = 0.006).

There were no significant differences between the SM and PM CL wearers in the number of participants with incorrect base curves (*X*^2(1)^ = 2.48, p = 0.12) and in the lens overall diameter (*X*^2(1)^ = 1.51, p = 0.22).

### Usage habits

Contact lens usage habits and CL fitting are detailed in Table 3. SM CL wearers were significantly more likely to use their lenses for more years, more daily hours, and tended to nap while wearing their lenses (p = 0.008, p<0.00001, *X*^2(1)^ = 5.09, p = 0.02, respectively). There was no significant difference between PM and SM CL wearers in washing hands before handling the CLs (*X*^2(1)^ = 0.57, p = 0.45), in changing the solution in the case every day (*X*^2(1)^ = 1.38, p = 0.24), and in frequency of case replacement (*X*^2(1)^ = 0.25, p = 0.62).

**Table 3:** Contact lens usage habits and fitting in contact lens wearers.

Contact lens usage habits and fitting		PM (N = 127)	SM (N = 132)	P value
**Years of CL wear**	**Mean ± SD**	5.4±4.6	6.4±4.1	0.008
	**Median** **(Q1, Q3)**	5 (2,7)	6 (4,8)	
**Mean daily hours**	**Mean ± SD**	10.0±3.7	12.0±2.9	<0.00001
	**Median** **(Q1, Q3)**	10 (8,12)	12 (10,15)	
**Napping/ sleeping with contact lenses**		29 (22.8%)	47 (35.6%)	0.02
**No handwashing before handling the contact lenses**		49 (38.6%)	57 (43.2%)	0.45
**Daily solution replacement**		31 (45.6%)	49 (55.1%)	0.24
**Case replacement every three months**		40 (51.3%)	48 (55.2%)	0.62

The contact lens usage habits of the professionally-managed (PM; first column) and self-managed SM; second column) with the p-values of the Kruskal-Wallis test that was employed to compare between the cohorts (third column). The number of participants that reported napping or sleeping with their contact lenses was a binary variable which was compared using the *X*^2^ statistic.

### Complications

The three cohorts differed significantly in their meibomian gland dysfunction grade (*X*^2(6)^ = 35.62, p<0.001), limbal redness (*X*^2(2)^ = 9.94, p = 0.007), corneal neovascularization (*X*^2(2)^ = 21.01, p<0.001), conjunctival staining (*X*^2(2)^ = 8.66, p = 0.01), and papillary conjunctivitis (*X*^2(2)^ = 24.05, p<0.001) (Table 4).

**Table 4:** Complications of control, non-contact lens wearers (NCL) professionally-managed (PM) contact lens wearers, and self-managed (SM) contact lens wearers included in the study.

Complications		NCL (N = 56)	PM (N = 127)	SM (N = 132)	P value
Meibomian gland dysfunction	0	35 (62.5%)	89 (70.1%)	49 (37.1%)	<0.001 Post-hoc: SM<NCL, p = 0.004 SM<PM, p<0.0001
	1	20 (35.7%)	28 (22.0%)	72 (54.5%)	
	2	1 (1.8%)	9 (7.1%)	11 (8.3%)	
	3	0 (0.0%)	1 (0.8%)	0 (0.0%)	
	4	0 (0.0%)	0 (0.0%)	0 (0.0%)	
Limbal redness		14 (25.0%)	40 (31.5%)	61 (46.2%)	0.007 Post-hoc: SM>NCL, p = 0.04 SM>PM, p = 0.04
Corneal neovascularization		7 (12.5%)	25 (19.7%)	53 (40.2%)	<0.001 Post-hoc: SM>NCL, p = 0.003 SM>PM, p = 0.003
Papillary conjunctivitis		16 (28.6%)	59 (46.5%)	87 (65.9%)	<0.001 Post-hoc: SM>NCL, p<0.0001 SM>PM, p = 0.005
Conjunctival staining		12 (21.4%)	16 (12.6%)	36 (27.3%)	0.01 Post-hoc: SM>PM, p = 0.01
Blepharitis		1 (1.8%)	10 (7.9%)	15 (11.4%)	0.09
Conjunctival redness		21 (37.5%)	39 (30.7%)	52 (39.4%)	0.33
Epithelial microcysts		0 (0.0%)	5 (3.9%)	2 (1.5%)	0.20
Corneal edema		0 (0.0%)	6 (4.7%)	8 (6.1%)	0.18
Corneal staining		16 (28.6%)	36 (28.3%)	43 (32.6%)	0.73
Corneal infiltrates		0 (0.0%)	4 (3.1%)	2 (1.5%)	0.33
Corneal ulcer		0 (0.0%)	7 (5.5%)	3 (2.3%)	0.11
Endothelial polymegathism/blebs		0 (0.0%)	2 (1.6%)	4 (3.0%)	0.36
Corneal distortion		2 (3.6%)	3 (2.4%)	7 (5.3%)	0.46
Superior limbic keratoconjunctivitis		6 (10.7%)	10 (7.9%)	14 (10.6%)	0.71

Comparison of 15 ocular complications in control, non-contact lens wearers (NCL), professionally-managed (PM) contact-lens wearers, and self-managed (SM) contact lens wearers. Meibomian gland dysfunction was graded on a scale ranging between 0–4 (Efron scale) and compared using the *X*^2^ statistic. Other abnormalities were graded on a binary scale (0-not present, 1-present) and compared using the *X*^2^ statistic (p-values presented in the last column with post-hoc Bonferroni comparisons presented below).

Post hoc (Bonferroni) analysis revealed that for all complications except for conjunctival staining, the scores of the SM CL wearers were worse than both the controls and the PM cohorts. Furthermore, for all complications except for conjunctival staining, the scores of the SM CL wearers did not differ significantly from the scores of the non-CL wearers. The meibomian gland dysfunction grade was significantly worse in the SM cohort compared to the control cohort and PM cohorts. Limbal redness was significantly worse in the SM CL wearers compared with both the PM CL wearers and non-CL wearers.

Corneal neovascularization was significantly worse in the SM cohort compared with both the control cohort and PM cohorts. Papillary conjunctivitis was significantly worse in the SM cohort compared with both the control and PM cohorts. Conjunctival staining was significantly worse in the SM cohort compared with the PM group.

### Subjective outcome measures

The control, PM and SM cohorts did not significantly differ in the number of participants that reported suffering from dry eye symptoms (26 (46.4%), 44 (34.6%), and 58 (43.9%), respectively; *X*^2(2)^ = 3.26, p = 0.19).

## Discussion

This study compared ocular complications, fitting parameters, usage habits, and dry eye symptoms of a cohort of soft CL wearers who are professionally-managed compared to a similar cohort who do not seek professional care. In order to assess if the complications are due to CL wear, and to examine if CL wear associated complications can be prevented with professional management, both CL wearing cohorts were also compared with control, non-CL wearers. Findings showed that the SM cohort had more ocular complications compared with both control and PM cohorts as well as a higher prevalence of inappropriate optical power when compared to the PM group. The SM cohort also significantly differed from the PM cohort in years of CL wear (6.42±4.07 vs. 5.43±4.55) and daily hours of CL wear (12.03±2.87 vs. 10.02±3.71). The mean difference in years of wear is one year on average (median: 5 vs. 6 years), which is not likely to account for the differences in ocular complications reported in this study. In addition, 13% more participants in the SM cohort reported sleeping or napping with their CLs, compared with the PM cohort (36% vs. 23%). This could indicate that non- compliance with recommended usage instructions could lead to increased ocular complications in the SM cohort and is in line with the conclusion of Rah et al. [25] that any CL, when used in a noncompliant manner, can lead to ocular complications.

The control cohort had significantly better VA than the SM CL wearers. Moreover, the PM and SM cohorts had a significantly higher refractive spherical component compared with the non- CL wearing control cohort. This finding is not surprising, given that approximately half of the control cohort wore spectacles (N = 26, 46%) and the other half did not require any refractive correction. The main objective for comparing with the control group from a separate study was to reveal which complications were due to CL wear. For example, limbal redness was 15% lower in its prevalence, corneal neovasculatization was 17% lower, and papillary conjunctivitis was 27% lower in the control, non-CL wearing cohort compared with contact-lens wearers. Although not reaching statistical significance, the prevalence of blepharitis in the control, non-CL wearing cohort was five times lower than in the CL wearers. These differences highlight the potential risks of CL wear. However, the comparison between three cohorts, also elucidates if the complications are more severe in SM CL wearers who are not under the care of a practitioner. Limbal redness, corneal neovascularization, and papillary conjunctivitis were significantly worse in the SM cohort compared with both the control non-CL wearing cohort as well as the PM cohort. These ocular complications were not significantly different in the PM and the control non-CL wearing control, emphasizing the potential importance of professional management of CL wearers whose ocular complications are more similar to non-CL wearers.

Papillary conjunctivitis was the most common complication among all of the CL wearers in this study, compared to the non-CL wearing controls (56% vs. 29%). This finding is similar to the results of both Sapkota et al. [26] and Kobia-Acquah et al. [27] who reported that papillary conjunctivitis was the most common ocular complication amongst 4063 CL wearers in Nepal (37%) and 117 CL wearers in Ghana (41%) respectively. A poorly fit CL can cause trauma to the tarsal conjunctiva and play a role in the development of papillary conjunctivitis [28]. Accordingly, in the current study, papillary conjunctivitis was 19% more prevalent in SM compared with PM CL wearers, and was also, unsurprisingly, significantly worse than found in the control, non-CL wearers.

The significantly higher prevalence of limbal redness in the SM cohort compared with the PM cohort could be attributed to fitting-related parameters as limbal redness has been linked with a poor contact lens fit and/or increased time of exposure of the anterior surface to a relatively hypoxic environment [29, 30].

Nomura et al. [31] previously reported a prevalence of 30% of neovascularization amongst 192 hydrogel CL wearers. Similarly, the present study found that the prevalence of corneal neovascularization among soft CL (55% hydrogel vs. 45% silicone hydrogel) wearers is 30%. This present study additionally compared complications in SM and PM contact lens wearers. Corneal neovascularization (NV) was two-fold more prevalent in SM compared with PM CL wearers, while the difference in NV prevalence between PM and control, non-CL wearers was only 7%. NV is known to be at least partially driven by corneal hypoxia, [32] and the amount of oxygen that reaches the cornea during CL wear depends on several factors such as CL wear time [31]. Our SM cohort reported an average two hours more daily CL wear, which could explain the higher probability of the development of blood vessels in their corneas.

In the SM cohort, 8.3% had a meibomian gland dysfunction grade of 2 or above, compared with 7.9% of the PM cohort. This difference, however, is not clinically meaningful. A previous investigation carried out by our laboratory also found that CL wearers did not differ significantly from control non-CL wearers in meibomian gland dysfunction grade [18]. Despite this finding, many more participants in the SM cohort had a meibomian gland dysfunction grade of 1 compared with the PM group (55% vs. 22%). A grade of 1 indicates a cloudy expression at some gland orifices and is not considered clinically worrying [3]. Over time, without treatment, however, grade 1 could develop into a higher grade, suggesting possibly increased MG complications in the future in the SM group.

One of the primary risk factors for conjunctival staining is lens fitting [33]. Accordingly, it is not surprising to us that the prevalence of conjunctival staining was found to be higher in the SM cohort compared with the PM cohort.

Several case reports also provide evidence of a higher complication rate when CLs are purchased through unregulated sources [34–36]. Unregulated supply of CLs is associated with several factors which could explain such an increased risk of complications. First, CLs are sold to the patient without any verification that the CLs provide an acceptable fit, nor that any subsequent CL wear effect on ocular physiology is within acceptable limits. Second, training in CL hygiene is not a normal component of unregulated CL supply. However, training may not be useful if CL wearers do not adhere to instructions. For example, Naaman et al. (2022) [37] reported that 41% of CL wearers were not compliant with lens case replacement. In the present study, a similar percentage of PM and SM CL wearers (49% and 45% respectively) were not compliant with CL case replacement. Further, Ramamoorthy and Nichols (2014) [38] reported that 52% of soft CL wearers did not wash their hands before handling lenses. In the present study, 39% and 43% of the PM and SM CL wearers reported not washing their hands before handling CLs. Despite this, eye care practitioners can remind CL users of the instructions during the follow-up visits, which is why the follow-ups can be considered important.

Third, without the supervision of a CL practitioner, there is also a likelihood of delay in seeking professional help in case of problems [39].

### Study strengths and limitations

The novelty of our present investigation is the comparison between SM and PM contact lens wearers, which is only possible in countries that do not regulate the sale of CLs with a prescription. The importance of the study is our documentation that there are more complications in SM contact lens wearers compared with PM contact lens wearers, which serves to emphasize the importance of professional CL fitting and follow-up.

One of the limitations of this study is the use of a control group from a separate study which varied from the PM and SM cohorts in a few characteristics. First, there was a significant difference between the number of female participants in the PM (82%) and SM (78%) cohorts compared to the control cohort (64%). CL wear is more prevalent in females in general which may explain why there were more females in the CL wearing cohorts compared with the non-CL wearing control cohort [40]. Second, there was a mean and median difference of two years between the control non-CL wearers cohort and the CL wearing cohorts. Although this difference was statistically significant, we believe that it is not clinically meaningful, as the incidence of notable meibomian gland abnormalities increases in the population above the age of 50 years [41]. Our cohorts included participants until the age of 45, and therefore the mean age difference between the cohorts is not likely to have affected the results of the study. Third, the control, non-CL wearing cohort was smaller as it was a group of participants from a different study. The fact that this group was not recruited at the same time as the CL wearing cohorts is also a limitation.

An additional limitation is that dry eye symptoms were assessed using a single question rather than a comprehensive standardized questionnaire, such as the Contact Lens Dry Eye Questionnaire-8 (CLDEQ-8), which is specifically designed for evaluating dry eye symptoms in soft contact lens wearers [42]. Previous research indicates that soft contact lens wearers exhibit a distinct pattern of symptoms compared to non-CL wearers [43].

A further questionnaire that can also be applied in future studies is the Contact Lens Discomfort Index (CLDI) [44] which is specifically intended to detect discomfort arising from CL wear, that was published after this study recruitment ended. Future studies can compare results of the CLDI questionnaire amongst PM and SM CL wearers.

## Conclusions

SM CL wearers were found to suffer from more ocular complications, tend to wear CLs with inappropriate parameters, and are less compliant with napping or sleeping with the CLs compared with PM CL wearers. Though the higher prevalence of some complications in the SM cohort was not clinically meaningful or large, these findings taken together highlight the importance of professional CL fitting, educating patients about proper contact lens use, and follow-up visits which reduces the risk of developing CL complications.

## Supporting information

S1 Appendix (DOCX)

S1 Data (XLSX)
