# Supplementary material for: Complications and compliance in professionally-managed and self-managed contact lenses compared with non-contact lens wearers
Source: PLoS One. 2024 Sep 6;19(9):e0308538. doi: 10.1371/journal.pone.0308538 (PMC11379192; doi:10.1371/journal.pone.0308538)
Supplement: S1 Appendix — (DOCX) [file pone.0308538.s001.docx]

**Appendix 1** Questionnaire

Full Name:

Date of Birth:

Sex:

Contact Lens History:

Contact Lens Type:

Last Eye Exam: □ Optometrist □ Ophthalmologist

- 3 months
- 6 months
- 12 months
- 24 months
- 36 months
- Four years or longer

Were the contact lenses fit by an optometrist or ophthalmologist

□ yes □ no

Was a slit lamp used in the fitting of the contact lenses (point to the slit lamp):

□ yes □ no

Where were the contact lenses purchased?

□ pharmacy □ optical □ internet □ other (please state):_______________

Do you sleep with your contact lenses at night?

□ never □ sometimes □ frequently □ all the time (very often)

Do you nap with your contact lenses?

□ never □ sometimes □ frequently □ all the time (very often)

Do you clean your lenses with the solution recommended by your eye care practitioner:

□ only what they recommended

□ the eye care practitioner did not recommend a specific solution

□ I use solutions that are on sale

□ I do not use cleaning solutions/ I use an alternative method to clean my lenses (please state):_______________

Which solution do you use: ____________________________

How often do you replace your lenses:

□ daily □ bi-weekly □ monthly □ annually □ other: _____

How many hours daily do you wear your lenses: _______________

Do you wash your hands before handling your lenses:

□ never □ sometimes □ frequently □ all the time

How often do you replace the solution in your contact lens case:

□ daily □ weekly □ occasionally

How often do you replace your contact lens case:

□ every month □ every 3 months □ every 6 months □ other:__________

For examiner to fill out:

Refraction

Right eye:

Left Eye:

Visual Acuity

Right Eye

Left Eye

MGD Efron Scale

Right Eye: ________________________

Left Eye: _________________________

Blepharitis

Right Eye:

□ yes □ no

Left Eye:

□ yes □ no

Limbal redness:

Right Eye

□ yes □ no

Left Eye

□ yes □ no

Conjunctival complications:

Right Eye

□ redness □ edema □ papillary conjunctivitis □ staining

Left Eye

□ redness □ edema □ papillary conjunctivitis □ staining

Cornea

Right Eye
□ edema □ ephithelial microcysts □ staining □ infiltrates □ ulcer
□ neovascularization □ endothelial polymegathism/ blebs □ distortions
□ superior limbic keratoconjunctivitis

Left Eye

□ edema □ ephithelial microcysts □ staining □ infiltrates □ ulcer
□ neovascularization □ endothelial polymegathism/ blebs □ distortions
□ superior limbic keratoconjunctivitis

Lens Fitting Parameters:

Right eye:

Base Curve

□ good □ flat □ steep

Overall diameter:

□ good □ small □ large

Optical Power: ______________________

Left eye:

Base Curve

□ good □ flat □ steep

Overall diameter:

□ good □ small □ large

Optical Power: ______________________
